# Selective blockade of microRNA-31-5p/calcitonin receptor interaction reverses established atrial fibrosis and atrial arrhythmia substrate

**DOI:** 10.21203/rs.3.rs-8484728/v1

**Published:** 2026-01-07

**Authors:** Jasha Trompf, Kathryn Cox, Mohit Hulsurkar, Lucia M Moreira, Chi Him Kendrick Yiu, Aaron M Johnston, Satadru K Lahiri, Shuai Zhao, Paul Robinson, Roddy Hiram, Mozhdeh Mehdizadeh, Lorena Perez Carrillo, Rana Sayeed, George Krasopoulos, Vivek Srivastava, Nicholas Walcot, Shakil Farid, Antonios Kourliouros, Priya Sastry, David A. Menassa, Benjamin Davies, Rita A Schack, Robia G Pautler, Keith M Channon, Craig Lygate, Stanley Nattel, Xander H T Wehrens, Svetlana Reilly

**Affiliations:** 1 Division of Cardiovascular Medicine, Radcliffe Department of Medicine, British Heart Foundation Centre of Research Excellence, University of Oxford, John Radcliffe Hospital; Oxford, OX3 9DU, UK.; 2 Cardiovascular Research Institute, Baylor College of Medicine; One Baylor Plaza, BCM335, Houston, USA.; 3 Department of Integrative Physiology, Baylor College of Medicine; One Baylor Plaza BCM335, Houston, USA.; 4 Faculty of Medicine, Department of Pharmacology and Physiology, and Research Centre, Montreal Heart Institute and University of Montreal; 5000 Bélanger Street Montréal (Quebec) H1T 1C8, Canada.; 5 Cardiothoracic Surgery, Oxford Heart Centre, John Radcliffe Hospital; Oxford, OX3 9DU, UK.; 6 Clinical Neurology, Nuffield Department of Clinical Neurosciences, University of Oxford, John Radcliffe Hospital; Oxford, OX3 9DU, UK.; 7 School of Cardiovascular & Metabolic Health, University of Glasgow; 126 University Place, Glasgow, G12 8TA, UK.; 8 Department of Pharmacology and Therapeutics, McGill University; 845 Sherbrooke St. West, Montreal, Quebec H3A 0G4, Canada.; 9 Institute of Pharmacology, West German Heart and Vascular Canter, University Duisburg-Essen; Hufelandstr. 55, 45122 Essen, Germany.; 10 IHU LIRYC and Fondation Bordeaux Université; 28 Avenue du Haut Leveque, 33600 Pessac, Bordeaux, France.

## Abstract

Atrial fibrillation (AF), the commonest cardiac arrhythmia, is a major contributor to mortality and morbidity. Atrial tissue fibrosis, a hallmark of structural remodelling in AF, is currently incurable and significantly hinders AF-treatment. *MicroRNA(miR)-31* is linked to ageing (a key risk factor for AF). Here, we show that AF-patients are characterised by upregulation of *miR-31–5p* in atrial cardiofibroblasts that negatively regulates the calcitonin receptor (*CTR*), thereby promoting atrial fibrogenesis and arrhythmia. Specific blockade of *miR-31–5p*/CTR-mRNA binding with LNA-miRNA-Target-Site-Blocker selectively increases atrial CTR expression and reverses advanced atrial fibrosis and arrhythmogenesis *in vivo*. These findings suggest a key role for *miR-31–5p*/CTR binding in promoting atrial fibrosis and arrhythmogenesis, and represents a first example of an RNA-based therapeutic capable of reversing established fibrosis that forms an AF substrate.

## INTRODUCTION

Atrial fibrillation (AF), the most common cardiac arrhythmia in humans, is a growing epidemic with an estimated incidence predicted to double by 2060, largely due to ageing. AF is a significant contributor to global mortality and morbidity, mainly due to stroke (AF alone is responsible for ~30–40% of all strokes) and heart failure (1, 2). Treatment of AF remains a major clinical challenge because of the underlying myocardial electrical and structural remodelling (3, 4). Electrical remodelling, caused by changes in ion channel expression/function, is largely reversible with maintenance of a normal sinus rhythm (SR) with the currently available anti-arrhythmic drugs, cardioversion and catheter ablation. By contrast, structural remodelling, hallmarked by atrial fibrosis, has not been successfully targeted by currently available therapies and is of particular concern in the clinic, as atrial fibrosis hampers AF treatment and worsens prognosis (5, 6). Thus, there is a quest to identify players in atrial fibrosis and cardiac remodelling to allow the development of more effective therapeutics for patients with this serious condition.^7^

Characterised by increased extracellular matrix (ECM) deposition without a significant loss of atrial cardiomyocytes (ACMs), atrial fibrosis increases as AF progresses (4) and is a key feature of cardiac remodelling in AF (8). The degree of atrial fibrosis negatively correlates with the success of one of the most successful therapeutic options, catheter ablation (9, 10). Despite extensive prior work, which uncovered key signalling pathways like those linked to transforming growth factor β1 (TGFβ1), galectin-3 and the renin-angiotensin-aldosterone system in atrial fibrosis, this insight has failed to yield clinically effective therapeutics to reverse atrial fibrosis. Therefore, there remains a major need to identify new players in atrial remodelling that can be targeted to generate more effective therapeutics for patients with AF (7, 9 10).

Atrial fibrosis is facilitated by complex interactions between cellular and neurohormonal mediators (11, 12), including a recently discovered cardioendocrine calcitonin (CT) system (13). This axis potently regulates atrial fibrogenesis and arrhythmia and is dysfunctional in the atria of patients with advanced atrial structural and electrical remodelling, such as in persistent-AF (persAF) (13). Cardiac CT produced by human ACMs controls proliferation, migration, and ECM production of atrial cardiofibroblasts (ACFs) (13). Disrupted signalling along this paracrine axis promotes collagen deposition, atrial fibrosis, and AF *in vivo* in mice. Patients with persAF have six-fold lower atrial CT production, reduced CTR expression and increased receptor internalization, facilitating enhanced collagen deposition by ACFs. Thus, restoring cardiac CT-CTR signalling represents an attractive new strategy to overcome major challenges in controlling cardiac remodelling in AF. While deficient CT can be replenished with exogenous CT-analogues (e.g. miacalcin, salmon CT) already in clinical use (e.g. for Paget’s disease, hypercalcaemia, osteoporosis), strategies to restore atrial CTR expression in persAF, a prerequisite for CT-mediated atrial anti-fibrotic and anti-arrhythmic effects, do not exist.

Given the reduced CTR protein in the absence of changes in CTR mRNA in ACFs from patients with persAF (13), it is plausible that microRNAs (miRs) may be involved in repressing CTR signalling. MiRs are endogenous small (19–27 nucleotides) non-coding RNAs which, via binding to a (partially) complementary sequence of 3’-untranslated mRNA region (3’UTR, or less commonly 5’UTR/CDs regions), mediate gene silencing by accelerating mRNA degradation or repressing translation (14). Previous work reported a number of miRs implicated in AF (15) including atrial-enriched *miR-31–5p* (16, 17), which, alongside three other miRs, is strongly predicted to bind the 3’UTR of the human CTR gene (*CALCR*). *MiR-31–5p* is implicated in ACM electrical remodelling in patients with persAF (17), in ageing (the strongest risk factor for AF (18)) and age-/senescence-related processes like cellular senescence, cancer and osteoporosis (19–21) (a condition associated with suppressed CT signalling). Our pilot work demonstrated that ACFs from patients with AF, which are characterised by supressed CTR-protein levels, produce twice the amount of *miR-31–5p*. Therefore, this study aimed to explore (i) the role of *miR-31–5p* as an upstream regulator of CTR signalling in remodelled atrial myocardium from patients with persAF, and (ii) the therapeutic potential of targeting the *miR-31–5p*/CTR mRNA interaction to prevent and/or reverse atrial fibrosis and arrhythmia.

## METHODS

### Patient cohorts.

Investigations using human samples were approved by the South Central-Berkshire B Research Ethics Committee (REF: 18/SC/0404). All patients gave informed written consent. A total of 136 patients were included in the study; all patients underwent cardiac surgery (coronary artery bypass or valve repair/replacement) in the John Radcliffe hospital, Oxford. Right and left atrial biopsies were collected during cardiac surgery, mostly before or shortly after (for LAA samples) cardiopulmonary bypass and immediately processed for cell isolation (described below) or snap-frozen until use in subsequent experiments (e.g., mRNA expression and immunoblotting).

### Animals.

Animal breeding, handling and experimental work were carried out in two centers, Baylor College of Medicine (Houston, USA) and the University of Oxford (UK) according to the local home office standards. All animal work was performed in accordance with the UK Home Office Animals (Scientific Procedures) Act 1986 incorporating Directive 2010/63/EU of the European Parliament, and in accordance with NIH guidelines (USA). Animals were kept in pathogen-free cages with controlled temperature and humidity. The total number of mice used in all animal studies was 175 consisting of 66 of *Lkb1*-aKD and 109 of *miR-31*/Cre mice. Animals of both sexes were used in the experiments.

### Generation of *Lkb1*-aKD mice.

The *Lkb1*-aKD mice were generated as previously described (13, 22). Briefly, AAV9-ANF-Cre was injected into 2–5 days old Lkb1^FL/FL^ pups (5×10^11^ GC, s.c. injections) generating atrial specific knockdown of *Lkb1* (*Lkb1*-aKD). Based on our previous studies (13, 22), these mice develop ectopic activities from week 5–6 that can be detected by surface ECGs. A 15-minute surface ECG was performed weekly to confirm ectopic activity and/or the onset of AF (from week 8 onwards). These mice were then treated with NC- or *miR-31*/CT-TSB either at 8 or 20 weeks (for the MRI cohort). Please see *Supplementary Materials* for more details.

### Generation of *miR-31*/Cre mice.

The *miR-31*/Cre mice were generated by PhiC31 integrase mediated cassette exchange in mouse embryonic stem cells to integrate a Cre recombinase-activatable *Pre miR-31* expression cassette at the *Gt(ROSA26)Sor* locus. Please see *Supplementary Materials* for more details.

### Isolation and culture of primary human atrial cardiofibroblasts (ACFs).

Human ACFs were isolated and cultured from atrial biopsies obtained from patients who underwent cardiac surgery, as previously described (13). Tissue biopsies were cut into small (2–3 mm^3^) pieces and repeatedly digested using 4 mg/ml collagenase II and trypsin (0.0625%), as previously described (13). Cells were washed twice with sterile phosphate-buffered saline (PBS) and plated onto 6-well plates in FBM-3 medium (#CC-3131, Lonza) containing 10% fetal bovine serum (FBS) and a supplement pack (#CC-4525, Lonza) and kept in a humidified atmosphere at 37 °C and 5% CO_2_. Please see *Supplementary Materials* for more details.

### Sources of other human cells.

In addition to the human primary cells, we also used HEK293 cells (#CRL-1573, ATCC) cultured and maintained according to the manufacturer’s instruction.

### Transfection of primary human atrial cardiofibroblasts (ACFs).

Human ACFs were transfected with *miR-31–5p* mimic, *miR-31–5p* inhibitor, or respective negative controls (NC-mimic, NC-inhibitor), or with *miR-31*/CTR-TSB and NC-TSB as previously described (16). Specifically, blockade of *miR-31–5p/CTR* interaction was achieved with 25 or 50 nM of antisense miRCURY LNA^™^ (locked nucleic acid) miRNA Power Target Site Blocker (TSB; #339199, Qiagen) G*G*G*C*A*A*G*A*G*G*T*A*A*T*C*T, or miRCURY antisense LNA^™^ Power Target Site Blocker negative control A (NC-TSB, #339199, Qiagen) A*C*G*T*C*T*A*T*A*C*G*C*C*C*A; LNA is not shown, as this information is proprietary. Cells were transfected using Lipofectamine RNAiMAX transfection reagent (#13778030, Invitrogen) in antibiotic-deprived FBM-3 medium containing 2 % FBS (Lonza).

Inhibition or overexpression of *miR-31–5p* was achieved by miRIDIAN *miR-31–5p* inhibitor (or NC-inhibitor) or mimic (or NC-mimic), respectively (all from Dharmacon: #IH-300507–06, IN-001005–01, C-300507–05 and CN-001000–01), using lipofectamine RNAiMAX transfection reagent (#13778030, Invitrogen) diluted in Opti-MEM medium (#31985062, Gibco), as described (16.) Efficient knockdown and overexpression was confirmed by real-time qPCR and visually by using the miRIDIAN Dy547-labeled transfection control (for experiments with *miR-31–5p* mimic or inhibitor). Please see *Supplementary Materials* for more details.

### Western blot.

Immunoblotting in human and murine atrial homogenates and in human ACFs was performed as described previously (13). Briefly, tissue or cell samples were lysed in RIPA buffer or CelLytic M cell lysis reagent, electrophoresis was performed using pre-cast 4–12% NuPAGE gels (#NP0335BOX, Invitrogen) and MOPS SDS running buffer (#NP0001, Invitrogen) at 150V. Proteins were transferred onto 0.2 μm nitrocellulose membrane (#1620112, Bio-Rad) using transfer buffer (#NP0006, Invitrogen). Immunodetection of the primary antibodies (Table 2, *Supplementary Information*) was performed using anti-IgG HRP-conjugated secondary antibodies (Table 2, *Supplementary Information*). Membranes were developed using a ChemiDoc System (BioRad, UK) and analysed with ImageJ software (version 1.52a) or Image Lab 6.1 (Bio-Rad). For stripping, nitrocellulose membranes were submerged in Restore PLUS Striping buffer (#46430, Thermo Scientific). The target protein and a loading control were always run on the same membrane.

### Colorimetric assays.

Quantification of total secreted collagen in the cell culture supernatant was performed using a Sirius Red collagen detection kit (#9049, Chondrex) as previously described (13). The amount of total collagen in murine atrial lysates was quantified by colorimetric detection of hydroxyproline using a Quickzyme total collagen assay kit (#QZBTOTCOL1, lot 0795, QuickZyme Biosciences).

### Scratch wound migration assay.

Human ACFs migration was determined using *in vitro* scratch wound assays as previously described (13). Scratch wound assay was performed on confluent monolayers of cells (following 24-hour transfection with *miR-31–5p* mimic, *miR-31–5p* inhibitor, or respective negative controls) using chambers with 2 well silicone insert with a defined cell-free gap (#80206, Ibidi). Please see *Supplementary Materials* for more details.

### Scar-in-a-jar assay.

Collagen 1 accumulation by fibroblasts was assessed using a scar-in-a-jar assay as in our previous work (13). Please see *Supplementary Materials* for more details.

### Assessment of cell proliferation.

Cell proliferation at a single time point was assessed using BrDU (5-Bromo-2’-Deoxyuridine) DNA-binding probe (#QIA58, Sigma-Aldrich) according to the manufacturer’s instructions and as previously described (13). Please see *Supplementary Materials* for more details.

### Assessment of gene expression with Real-Time Quantitative Polymerase Chain Reaction (qPCR).

As previously described (13, 16), atrial tissue samples were homogenised using an electric homogeniser Polytron with an appropriate lysis buffer. Total RNA was extracted with Nucleospin RNA II kit (#740955.250, Macherey-Nagel) following manufacturer’s instruction. Complementary DNA (cDNA) was synthesised from 250 ng of RNA with high-capacity cDNA reverse transcription kit (#4368814, Thermofisher Scientific) according to the manufacturer’s protocol. Total RNA from human ACFs was extracted with mirVana miRNA isolation kit (#AM1561, Invitrogen). cDNA was synthesised from 100 ng RNA using QuantiTect Rev. Transcription Kit (#205313, Qiagen). For *miR-31–5p/3p* reverse transcription (RT), the TaqMan MicroRNA Reverse Transcription Kit (#4366596, Applied Biosystems) and TaqMan MicroRNA assay RT primers were used. Please see *Supplementary Materials* for more details.

### RNA-binding protein immunoprecipitation (RIP) with Ago2 in human ACFs.

Human ACFs from patients with persAF were transfected with *miR-31*/CTR-TSB or NC-TSB (25 nM) for 24 hours. The RIP assay was performed using the Magna RIP kit (#17–700, Sigma-Aldrich) as previously described (16). In brief, cells were washed in ice-cold PBS (two times) and lysed in 100 μl complete RIP lysis buffer. Magnetic beads were conjugated with 5 μg of anti-Ago2 antibody (#ab32381, Abcam) and subsequently incubated with the RIP lysates overnight at 4°C with rotation. Immunoprecipitates were purified and kept overnight at −80°C to precipitate the RNA and resuspended in RNase-free water. RNAs were subjected to RT-qPCR to measure the *CALCR* mRNA and *miR-31* levels. *CALCR* gene expression was normalised to *miR-31* and quantified using the comparative threshold cycle method (2^−ΔCt^). Please see *Supplementary Materials* for more details.

### Transcription inhibition with actinomycin D.

Human ACFs from patients in SR were transfected with 25 nM of miRIDIAN *miR-31–5p* mimic or NC-mimic for 24 hours and then treated with 5 μg/ml actinomycin D (#A9415, Sigma-Aldrich) for ~8.5 hours. Levels of *CALCR* (qPCR) are shown as percentage of the respective mRNA (2^−ΔCt^) at 0 hour, normalising to *GAPDH* as the housekeeping gene. Please see *Supplementary Materials* for more details.

### Dual-Glo firefly luciferase reporter assay in HEK293 cells.

The reporter construct was generated using the pmirGLO Dual-Luciferase miRNA Target Expression Vector (#E1330, Promega) containing the whole length (1869 bp) of *CALCR* 3′UTR. Please see *Supplementary Materials* for more details.

### Histological assessment of cardiac fibrosis with Masson’s trichrome staining in mice.

Staining for collagen was performed using Masson’s trichrome staining as described before (13, 22). All experiments and analysis were performed blinded. Please see *Supplementary Materials* for more details.

### Transthoracic echocardiography.

Cardiac functions of the *Lkb1*-aKD, *miR-31*/Cre mice and control mice were assessed with transthoracic echocardiography. Please see *Supplementary Materials* for more details.

### *In vivo* transesophageal burst pacing in *miR-31*/Cre and WT/Cre mice.

AF inducibility in *miR-31*/Cre and WT/Cre mice was assessed with transesophageal burst pacing as described previously (16, 23). Please see *Supplementary Materials* for more details.

### Telemetry studies.

Telemetry studies were performed as described previously (26). Please see *Supplementary Materials* for more details.

### MRI in mice.

Mice underwent MRI before *miR-31*/CTR-TSB or NC-TSB treatment and 3.5 weeks later. All MRI imaging data were acquired using a Bruker Biospec 9.4 T, 20 cm bore, AV NEO/Paravision 360 at the Small Animal Imaging Facility at Texas Children Hospital. Please see *Supplementary Materials* for more details.

### *In vivo miR-31*/CTR-TSB administration in mice.

Blockade of the *miR-31*/CTR interaction was achieved with antisense miRCURY LNA miRNA Power TSB (#339202, Qiagen) G*G*G*C*A*A*G*A*G*G*T*A*A*T*C*T, or NC-TSB (#339202, Qiagen); LNA is not shown, as this information is proprietary. Please see *Supplementary Materials* for more details.

### A sandwich hybridization assay of LNA-TSB.

A sandwich hybridisation assay was performed using a biotinylated LNA-capture probe complementary to half of the LNA-*miR-31*/CTR-TSB to capture the LNA-TSB on streptavidin-coated magnetic beads. Please see *Supplementary Materials* for more details.

### RNA sequencing: sample processing and analysis.

Total RNA was extracted from hACF cell cultures using *mirVana* miRNA Isolation Kit (Thermo: AM1560) as above. Please see *Supplementary Materials* for more details.

### Statistical analysis.

The data distribution was tested by Kolmogorov-Smirnov or Shapiro-Wilk tests. The unpaired or paired Student’s *t* test was used in two-group comparisons of the normally distributed data. Multiple groups of normally distributed data of similar variance were compared by one-way or two-way ordinary or repeated measures ANOVA with Holm-Sidak’s or Sidak’s correction. When the normality assumption was not met the data were analysed by Mann-Whitney or Wilcoxon test - for independent or paired comparisons between 2 groups, respectively – or Kruskal-Wallis with Dunn’s correction - for multiple group comparisons. Categorical variables were compared by Fisher’s exact test. All statistical analysis was performed using GraphPad Prism v7, v6.04, v9.0 and v10 software. A value of P < 0.05 was considered statistically significant. Pearson’s correlation test was used to test correlation between 2 normally distributed variables.

### Data availability:

**A**ll data generated or analysed during this study are included in the article. The RNAseq data is deposited REF: GSE294333 at https://www.ncbi.nlm.nih.gov/geo/query/acc.cgi?acc=GSE294333

## RESULTS

### *MicroRNA-31–5p* regulates CTR abundance in human ACFs.

**Fig. 1.**
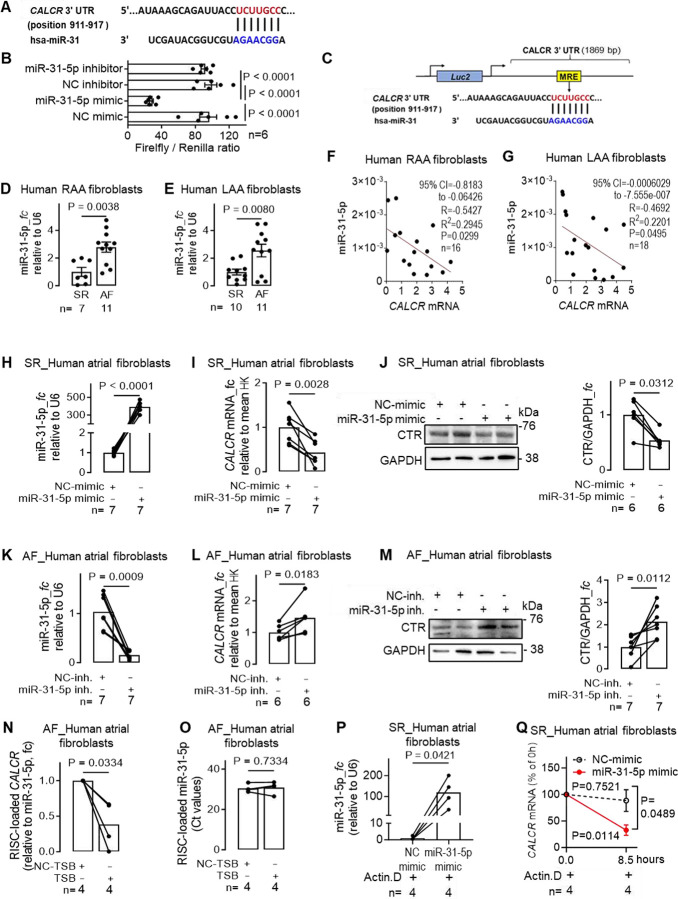
Atrial-specific *miR-31–5p* controls CTR expression in human atrial fibroblasts. **(A)** *In silico* predicted miRNA-recognition element (MRE) between *hsa-miR31–5p* and 3’UTR of CTR mRNA (*CALCR)*. **(B)** Luciferase reporter assay in HEK293 cells transfected with miR-31 mimic, *miR-31–5p* inhibitor, or respective negative controls (NC-mimic, NC-inhibitor). **(C)** Diagram of the luciferase reporter construct used in (B). **(D-E)** Level of *miR-31–5p* expression in RAA (D) and LAA (E) human atrial cardiofibroblasts (ACFs) collected from patients with persistent atrial fibrillation (AF) vs control in sinus rhythm (SR). **(F-G)** Correlation between *miR-31–5p* and *CALCR* in human RAA (F) and LAA (G) ACFs. **(H-M)** Effect of miR31 overexpression (H) or inhibition (k) on CTR gene (I and L) and protein (J and M) expression in human atrial fibroblasts. **(N-O)** Prevention of miR-31 binding to *CALCR* with MRE-specific *miR31*/CTR-TSB (vs NC-TSB) reduces loading of *CALCR* (N) into RNA-induced-silencing-complex (RISC), without altering *miR-31–5p* RISC loading (O). **(P-Q)** Effect of *miR-31–5p* overexpression (N) or NC-mimic on *CALCR* (normalised to a 0 hour-control) in human SR-ACFs (Q) pre-treated with actinomycin D (Actin.D); p-value shows interaction for miR-31 mimic treatment. Data are expressed as mean ± SEM, except median with IQR in (J). P-values calculated by 1-way ANOVA with Holm-Sidak’s test (B), unpaired t test (D, E), Pearson correlation test (F, G), paired t test (H, I, K-P), Wilcoxon test (J), or by two-way RM ANOVA (Q). fc, fold change of control group; n, individual donors.

Using published reports (**table S1**) of the miRs altered in atrial tissue or cells in AF, we identified, using *in silico mirDIP* analysis at https://ophid.utoronto.ca/mirDIP/, four miRs (*hsa-miR-24–3p*, -* 30d-5p*, *−31–5p* and *34a-5p*), which are strongly predicted to bind CTR-mRNA (*CALCR*) 3’UTR (**fig. S1A**). A reporter assay showed no binding between *CALCR*-3’UTR and *hsa-miR-24–3p*, *−30d-5p*, or *−34a-5p* in HEK293 cells co-transfected with pmiRGLO luciferase/renilla-expressing plasmid containing full-length *CALCR*-3’UTR and the respective miR mimic/inhibitor (vs mimic/inhibitor-non-targeting negative controls [NC]) (**fig. S1B–H**). By contrast, the reporter assay showed ~60% reduction of the luciferase/renilla ratio (P=0.001) in cells transfected with *miR-31–5p* mimic (*vs* NC-mimic) that was fully reversed by *miR-31–5p* inhibition, indicating that *miR-31–5p* binds the *CALCR*-3’UTR (fig. 1A–C). While upregulation of *miR-31–5p* has been reported in human ACMs and noted to be relevant to electrical remodeling in AF (16, 17), its role in ACFs and its potential contributions to AF fibrogenesis is unknown. We found that in patients (**table S2**) with persAF, compared to SR controls, ACFs from both right (RAA; P=0.003) and left (LAA; P=0.008) atrial appendages had 2.7- and 2.5-fold increased *miR-31–5p* expression respectively (fig. 1D, E); notably, miR-31–5p expression was barely detectable in the left ventricular myocardium (fig. S1I). Moreover, *miR-31–5p* levels negatively correlated with *CALCR* expression in these ACFs (RAA: P=0.029; LAA: P=0.049; fig. 1F, G). To test the effect of miR-31–5p on CTR gene and protein expression in native ACFs, ACFs were isolated from SR and persAF patients were transfected with *miR-31–5p* mimic (or NC-mimic) and *miR-31–5p* inhibitor (or NC-inhibitor) respectively. Overexpression of *miR-31–5p* (fig. 1H) halved CTR-mRNA (P=0.0028) and protein (P=0.0312) (fig. 1I, J), while *miR-31–5p* inhibition (fig. 1K) increased CTR-mRNA by ~30% (P=0.0183) and nearly doubled CTR protein (P=0.017) *vs* NC-treated ACFs (fig. 1L, M). These results indicate that *miR-31–5p* inhibition is sufficient to restore the decreased ACF-CTR expression previously observed in persAF (13).

To confirm the endogenous interaction between *miR-31–5p* and *CALCR* (i.e., occupancy of miR-31–5p on *CALCR-3’UTR*) we assessed, with previously validated RNA-binding protein immunoprecipitation (RIP) assay and qPCR (16), the loading of both targets into the RNA-Induced-Silencing-Complex (RISC) in AF-ACFs. While both *CALCR* and *miR-31-*5P were present in Ago2-RISC pulldowns (fig. 1N, O), a selective blockade of the *miR-31–5p*/*CALCR* interaction with LNA-power-Target-Site-Blocker (miR-31/CTR-TSB) decreased *CALCR*, but not *miR-31–5p,* RISC content by ~60% (P=0.0334) (fig. 1N, O), further confirming an endogenous *miR-31–5p*/*CALCR* interaction in human AF-ACFs. To elucidate the mechanism (enhanced mRNA degradation or translational silencing) by which *miR-31–5p* regulates *CALCR*, we compared the effects of *miR-31–5p* mimic (or NC-mimic) (fig. 1P) on *CALCR* levels in human ACFs (hACFs) pre-treated with 5 μg/ml actinomycin D, a potent, non-selective transcription inhibitor (17); *CALCR* diminished faster in the presence of *miR-31-5p* mimic (vs NC-mimic) (P=0.0114), pointing towards enhanced mRNA degradation as the predominant mechanism by which *miR-31–5p* regulates CTR expression in hACFs (fig. 1Q).

### *MicroRNA-31–5p* regulates CTR expression in atrial fibroblasts in mice.

**Fig. 2.**
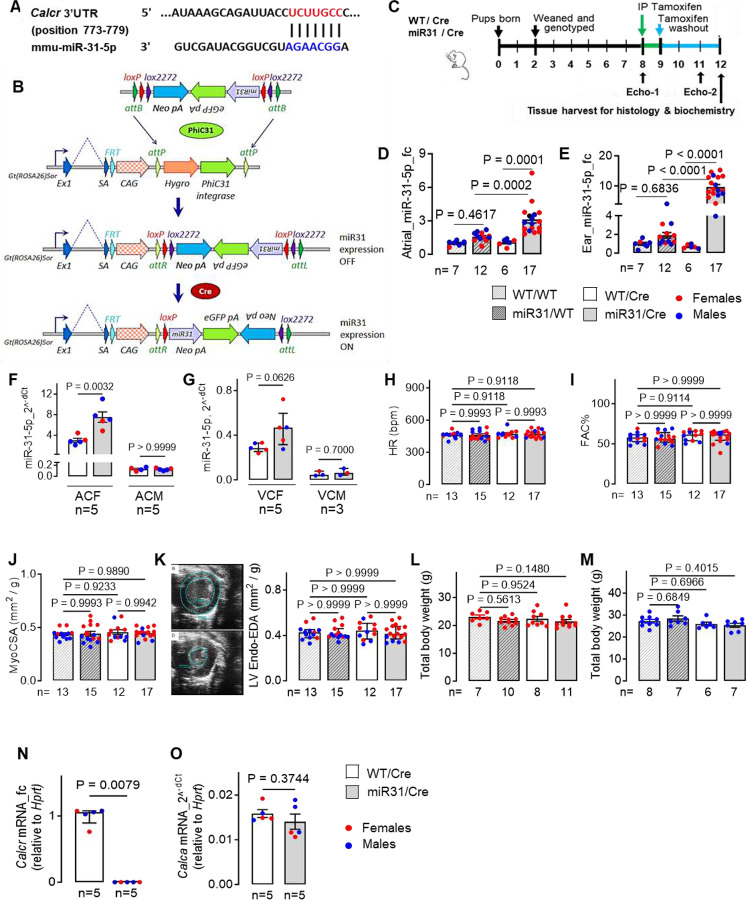
Induced fibroblast-targeted miR-31 overexpression inhibits CTR expression in mice. **(A)** *In silico* predicted miRNA-recognition element (MRE) between *mmu-miR31–5p* and 3’UTR of CTR-mRNA (*Calcr*). **(B)** Schematic representation of the constructs used to generate fibroblast-specific miR31/Cre mice. **(C)** Experimental timeline for miR31/Cre mice studies. **(D-E)** Atrial tissue (D) and ear (E) gene expression of *mmu-miR31* in all genotypes in male (in blue) and female (in red) mice. **(F-G)** Upregulation of miR-31 in atrial (F) and left ventricular (G) cardiofibroblasts (ACFs, VCFs) and cardiomyocytes (ACMs, VCMs) following Cre recombination. h, Heart rate in all four mouse genotypes. **(I-K)** Echocardiography showing unaltered fractional area of change (FAC), myocardial cross-sectional surface area (MyoCSA); and *endocardial* end-diastolic area (LV-Endo-EDA) between all four mouse genotypes. Short-axis images in (K) are examples of end-diastolic view of the end-diastolic endocardial and epicardial areas (top panel), and short-axis echocardiograms of the endocardial end-systolic area – bottom panel); data in (J) and (K) normalised to total body weight. **(L-M)** Total body weight of females (L) and males (M). **(N-O)** CTR (*Calcr*) and CT gene expression (*Calca*) in atrial fibroblasts and cardiomyocytes (qPCR) respectively in miR-31/Cre and WT/Cre mice. Data are expressed as mean ± SEM, or median with IQR (G, I, K, L, O, R, S). P-values calculated by one-way ANOVA with Holm-Sidak’s (D, E, H, J) or Dunn’s post-hoc test (M), Kruskal-Wallis with Dunn’s post-hoc test (I, K, L), unpaired t test (F - ACF group; O), Mann-Whitney test (F - ACM group; G, N); fc, fold change of control group; n, individual donors.

Similar to humans, the mouse CTR (*Calcr*) mRNA also contains a conserved *miR-31–5p* binding site in its 3’UTR (fig. 2A). To test the effect of *miR-31–5p* /*Calcr* binding *in vivo*, we generated a tamoxifen-inducible fibroblast-targeted *miR-31* transgenic mouse model (miR31/Cre) using *Col1a2-*Cre-ER(T) transgene (fig. 2B, C; **fig. S2, S3A)**. A qPCR confirmed successful overexpression of murine *miR-31–5p* in atrial and ear (a positive fibroblast-enriched control) tissue lysates (fig. 2D, E) in miR31/Cre mice vs control WT/Cre, or other relevant genotypes (WT/WT and miR31/WT), in both female and male animals. The *miR-31–3p* strand, which does not have a binding site for CTR-mRNA-3’UTR, was also (though to a lesser extent) upregulated (by ~30%, **fig. S3B** compared to ~2-fold for *miR-31–5p*, fig. 2F), in murine ACFs. Analysis of *miR-31–5p* expression in isolated atrial and ventricular cardiofibroblasts (CFs) and cardiomyocytes (CMs) showed a 2.5- and 1.6-fold increased expression in atrial-CFs (P=0.003) and ventricular-CFs (VCFs), though it did not reach statistical significance in VCFs (P=0.062), with unchanged levels in atrial-CMs (P>0.999) and ventricular-CMs (P=0.700) (fig. 2F, G). These results indicate the successful overexpression of the *miR-31* transgene CFs. Neither *miR-31*-transgene nor tamoxifen administration affected ventricular function in mice in either genotype in males or females (fig. 2H–M, **S4A-H; table S3, S4, S5**). Similar to hACFs, murine *miR-31* overexpression significantly decreased atrial CTR protein and atrial fibroblast mRNA (fig. 2N, **S5A**) compared to the control WT/Cre animals. This was associated with unchanged expression of the CTR ligand calcitonin (CT) transcript (*Calca*) expression in murine ACMs (fig. 2O).

### Effect of *miR-31–5p* on hACF functions.

**Fig. 3.**
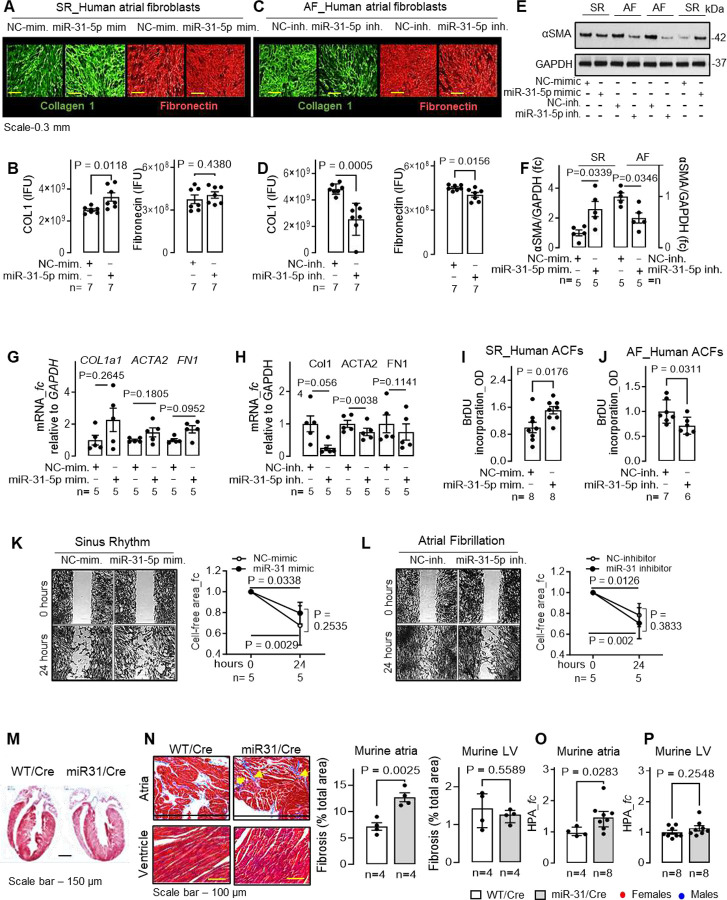
*MiR-31* exacerbates profibrotic responses in human atrial fibroblasts and promotes atrial fibrosis in vivo in mice. **(A-D)** Effect of *miR-31* overexpression (A, B) or inhibition (C, D) on collagen and fibronectin production (scar-in-a-jar) in human atrial cardiofibroblasts (ACFs) obtained from control patients in SR (A, B) and persistent AF (persAF) (C, D). Scale bar - 0.3 mm. **(E-F)** Representative blots (E) and quantification (F) of α-smooth muscle actin (αSMA) protein in ACFs obtained from patients with SR and persAF transfected with miR-31 mimic and inhibitor respectively. **(G-H)** Gene expression (qPCR) of collagen 1 (*COL1*), αSMA (*ACTA2*) and fibronectin (*FN1*) in human ACFs transfected with *miR-31* mimic and inhibitor in SR (g) and AF (h) groups respectively. **(I-L)** Effect of *miR-31* mimic and inhibitor on proliferation (BrDU assay; i-j), cell migration (scratch assay, K-L) in human SR- and AF-ACFs. **(M-P)** Effect of induced miR-31 overexpression on atrial and ventricular fibrosis assessed by Masson’s trichrome staining (M, N) and hydroxyproline (HPA) assay (O, P) in miR31/Cre and WT/Cre mice. Data are expressed as mean ± SEM, except median with IQR in (H - for COL1a1; O). P-values calculated by unpaired t test (B, D, I, J, N, P), paired t test (F, G, H - for ACTA2 and FN1), Mann-Whitney test (O,), Wilcoxon test (H - for COL1a1), two-way RM ANOVA with Sidak’s test (K, L); fc, fold change of control group; n, individual donors; mimic - mim.; inhibitor - inh.

To evaluate the functional responses of hACFs to *miR-31* overexpression or inhibition, we performed *in vitro* studies in these cells. ECM collagen-1 accumulation (assessed by scar-in-a-jar assay) was ~30% increased (P=0.0118) by 72-hour overexpression of *miR-31–5p* mimic in SR-hACFs (fig. 3A, B). By contrast, inhibition of *miR-31–5p* with *miR-31–5p* inhibitor halved collagen-1 content in ACFs from patients with persAF (fig. 3C, D). Similar effects were obtained in the presence of TGFβ1 stimulation (5 ng/ml, **fig. S4I, J**). *MiR-31–5p* mimic had no effect on fibronectin protein in hACFs (fig. 3A–D) – possibly due to a maximal activation of cell responses in persAF. Expression of α-smooth muscle actin (αSMA) increased 2-fold (P=0.0339) in *miR-31–5p* overexpressing SR-hACFs, while it was halved (P=0.0346) by *miR-31–5p* inhibition in AF-hACFs (fig. 3E, F). While overexpression of *miR-31–5p* did not alter transcript expression of *COL1, ACTA2* and *FN1*, miR-31–5p inhibition reduced *ACTA2* (P=0.0038) with a trend towards a reduction for *COL1* (P=0.056) and unchanged *FN1* in hACFs (fig. 3G, H). Cell proliferation was 50% higher (P=0.0176) in *miR-31–5p* mimic SR-hACFs (fig. 3I) and reduced by ~29% (P=0.0311) by *miR-31–5p* inhibition in AF-ACFs (fig. 3J). However, it remained unchanged in TGFβ1-activated hACFs in both *miR-31–5p* mimic or inhibitor treated groups (**fig. S4K, L**), likely attributable to maximal ACF activation and proliferation response to TGFβ1. Neither *miR-31–5p* mimic (vs NC-mimic; fig. 3K) nor *miR-31–5p* inhibitor (vs NC-inhibitor; fig. 3L) affected the migration of hACF. These results suggest that overexpression of *miR-31–5p in vitro* is sufficient to activate some key fibrotic responses (collagen-1 and αSMA production, and cell proliferation) in hACFs which, in the presence of persAF, are significantly suppressed by *miR-31–5p* inhibition.

### Upregulation of *miR-31* in fibroblasts promotes structural and arrhythmogenic substrate *in vivo*.

**Fig. 4.**
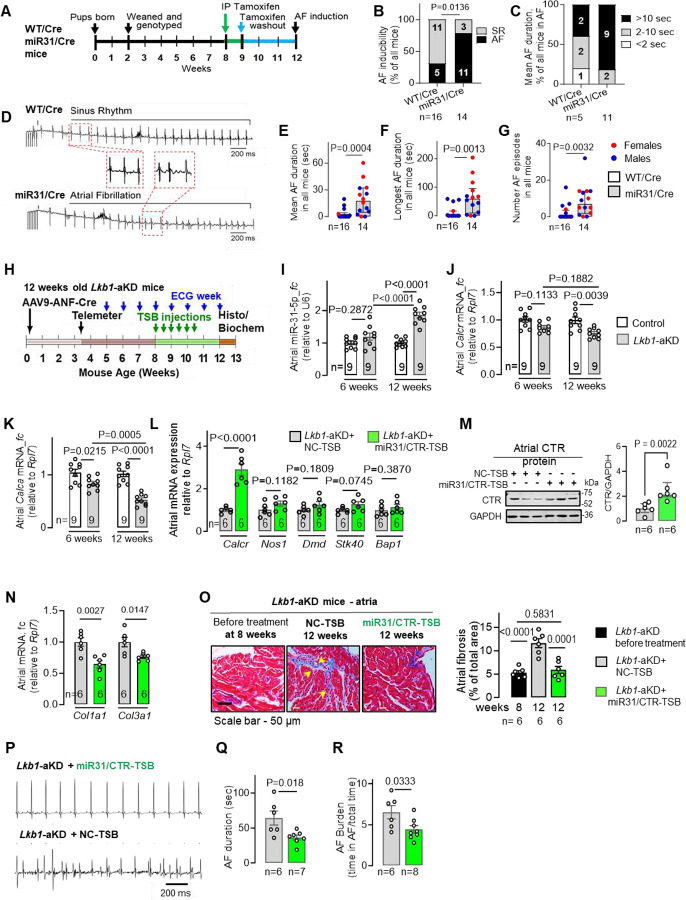
*MiR-31* overexpression induces atrial fibrosis and increases AF susceptibility i*n vivo* in mice prevented by targeted disruption of the *miR-31*/*Calcr* interaction. **(A)** Experimental timeline in *miR-31*/Cre mouse studies. **(B-G)** Atrial burst pacing-induced AF (B; % of all mice), the mean (C, E) and longest (F) AF episode duration and, the number of AF episodes (G) in *miR-31*/Cre vs Cre/WT mice; example of the typical ECG recording is in (D). **(H)** Experimental timeline in 12-week old Lkb1-aKD mice. **(I-K)** Changes in atrial tissue *miR-31–5p*, CTR (*Calcr*), and CT (*Calca*) mRNA expression in Lkb1-aKD (*vs* controls) mice at 6 and 12 weeks. **(L)** Effect of miR31/CTR-TSB on mRNA of CTR and previously validated *miR-31* targets, *Nos1,* dystrophin *(Dmd), Stk40 and Bap1* in murine atrial tissue. **(M)** Effect of *miR-31*/CTR-TSB on atrial CTR protein in 12-week old *Lkb1*-aKD mice. **(N)** Effect of *miR-31*/CTR-TSB or NC-TSB on atrial expression of collagens, *Col1* and *Col3a1,* in 12-week old *Lkb1*-aKD mice. **(O)** Atrial fibrosis (by Masson’s trichrome staining) in 12-week old *Lkb1*-aKD mice treated with *miR-31*/CTR-TSB or NC-TSB control. **(P-R)** Representative ECG recordings (P), the AF episodes, the duration of the longest AF episodes recorded by ECG telemetry (Q) and AF burden (R) in *Lkb1*-aKD mice treated with miR31/CTR-TSB or NC-TSB. Data are expressed as mean ± SEM, except median with IQR in (E-G, N - for *Col3a1*). P values are calculated by unpaired t test (K, L, N, Q, R, N - except *Col3a1*), except for the one-sided Fisher’s exact test (B), Mann-Whitney test (E-G, N - for *Col3a1*), one-way ANOVA (I, J, K, O); fc, fold change of control group; n, individual donors.

To test whether the atrial fibrotic remodelling in persAF caused by the loss of CTR is secondary to *miR-31* upregulation, we used the fibroblast-specific *miR-31*-overexpressing mice (fig. 2). These mice displayed a doubled (P=0.0025) amount of atrial (but not ventricular) fibrosis assessed by Masson’s trichrome staining (fig. 3M, N) and hydroxyproline assay (P=0.028) (fig. 3O, P) compared to the WT/Cre controls. These alterations were accompanied by ~40% increase in atrial protein levels of collagen-1 (P=0.016), collagen-3 (P=0.006) and αSMA (P=0.049) (**fig. S5A, B, C**), while mRNA was unchanged for collagen-1, increased for collagen-3 and reduced for αSMA (**fig. S5D-F**). Atrial fibronectin protein remained unchanged (despite decreased its mRNA; P=0.0429; **fig. S5A, G**). By contrast, left ventricular collagen-1, collagen-3 and fibronectin proteins remained unaltered, while αSMA increased (**fig. S5H, I**). Overexpression of *miR-31* also caused a reduction in the mRNA (**fig. S5J-M**) and, in some cases, protein (with the exception of BAP1 - remained unchanged, and TNS1 - increased) expression in the atria of previously validated *miR-31*-targets, *FGF-1, BAP1, STK40* and *TNS1* (**fig. S5A, B**). Increased atrial fibrosis in mice was associated with a 2-fold higher AF inducibility (P=0.0136) (fig. 4A, B), prolonged AF duration (fig. 4C–F) and more frequent AF episodes (fig. 4G). As *miR-31* overexpression in mice did not differ between males and females and had no correlation with sex, and there were no sex-dependent differences in heart rate, ventricular function or AF duration/number of AF episodes, we did not analyse males and female results separately in the downstream experiments. Our findings suggest that a clinically relevant 2-fold upregulation of *miR-31* in atrial fibroblasts (fig. 2F) is sufficient to promote the formation of an AF substrate *in vivo*.

### *MiR-31–5p* alters the transcriptional programme of hACFs.

To assess whether *miR-31–5p* alters other potential target genes and the broader transcriptional programme of hACFs, control SR-ACFs were transfected with *miR-31–5p* mimic, and AF-ACFs with *miR-31–5p* inhibitor (or respective NC-controls), and submitted for bulk RNA-sequencing (RNAseq) (**fig. S6**). Principal component analysis (PCA) revealed clustering of different groups by treatment and a distinct pattern in differentially expressed genes (DEGs) between *miR-31–5p* mimic and inhibitor vs NC-mimic/NC-inhibitor groups (**fig. S6A-D**). While *miR-31–5p* overexpression significantly upregulated 1023 and downregulated 1009 genes, *miR-31–5p* inhibition upregulated 70 and downregulated 70 genes (**fig. S6C, D; data S1**). RNAseq and qPCR confirmed concordant changes in previously reported *miR-31* gene targets, *BAP1, PKN2* and *STK40* (24, 25), in hACFs transfected with *miR-31–5p* mimic and inhibitor (**fig. S6E-N**). DEG pathway analysis found that *miR-31–5p* overexpression leads to the activation of microtubule movement/formation, as well as pathways involved in cilium organisation and assembly (**fig. S6O)**, while inhibiting cell cycle and nuclear chromosome organisation and function (**fig. S6P**). These transcriptional changes may contribute to the *miR-31*-mediated fibrotic responses in AF-hACFs (26). By contrast, *miR-31–5p* inhibition in AF-hACFs alters genes involved in TGFβ, TNF and steroid hormone signalling (fig. **S6Q**). Notably, *miR-31–5p* mimic treatment elevated the expression of several fibrosis-relevant genes (e.g. *BMP1, COL3, FAP, FN1, POSTN and THBS1*; **fig. S6R**), the relevance of which should be explored in future work. *MiR-31–5p* inhibition was also associated with suppressed inflammation-related DEGs, *CCL5, CCL26, TNFAIP6, IFI6* and *HSPA6/7* (**fig. S6S**). The concordant changes between *miR-31–5p* and these DEGs suggest an indirect effect of *miR-31–5p* on them.

### Selective disruption of *miR-31–5p*/*CALCR* interaction reverses atrial fibrosis and arrhythmia *in vivo*.

LNAs are modified nucleic acids with enhanced binding affinity for their target RNA (27). Using miRCURY LNA-miR-TSB *in vivo* to selectively block the *mi-R31–5p*/*CALCR* interaction, we tested causality between the *miR-31–5p*/*CALCR* interaction, atrial fibrosis, and AF development in the previously-validated *Lkb1*-aKD mouse model of spontaneous AF (fig. 4H) (13, 22). Similar to the changes observed in patients with persAF (13) (fig. 1B–E), the *Lkb1*-aKD mice displayed almost doubled levels of *miR-31–5p* (fig. 4I), and reduced (by ~30%) *Calcr*- and *Calca*-mRNA (fig. 4J, K) (vs Lkb1^FL/FL^ animals) at 12 (and for *Calca* as early as 6) weeks of age. Thus, *Lkb1*-aKD mice represent a clinically relevant model in the context of the AF-associated activation of *miR-3–5p1*/CTR signalling.

We initially injected 8-week-old *Lkb1*-aKD mice with *miR-31*/CTR-TSB or respective NC-TSB and monitored structural remodelling (atrial fibrosis) and AF burden/duration for 4 weeks. As 8-week-old mice do not exert established fibrosis and structural remodelling, they were used to test preventive value of the TSB treatment on structural remodelling and arrhythmogenesis. We observed that *miR-31*/CTR-TSB treatment rescued atrial CTR-mRNA (P=0.0001) and protein (P=0.0076) (fig. 4L, M) without changes in other previously validated targets of miR-31 (*Nos1*, dystrophin (*Dmd*), *Stk40* and *Bap1*; fig. 4L), thus confirming the specificity of the TSB approach to disrupt the *miR-31–5p*/*Calcr* interaction. Compared to NC-TSB, the *miR-31*/CTR-TSB treatment suppressed expression of the atrial collagen-1 (by ~40%, P=0.0027) and collagen-3 (by ~25%, P=0.0147) (fig. 4N), prevented the development of atrial fibrosis (P=0.0001) assessed with Masson’s trichrome stain (fig. 4O), and reduced AF duration by ~45% (P=0.018) and burden by ~32% (P=0.0333) (fig. 4P–R). Therefore, selective blockade of *miR-31*/*CALCR* binding is sufficient to preclude atrial fibrogenesis and control atrial arrhythmogenesis *in vivo* in mice.

**Fig. 5.**
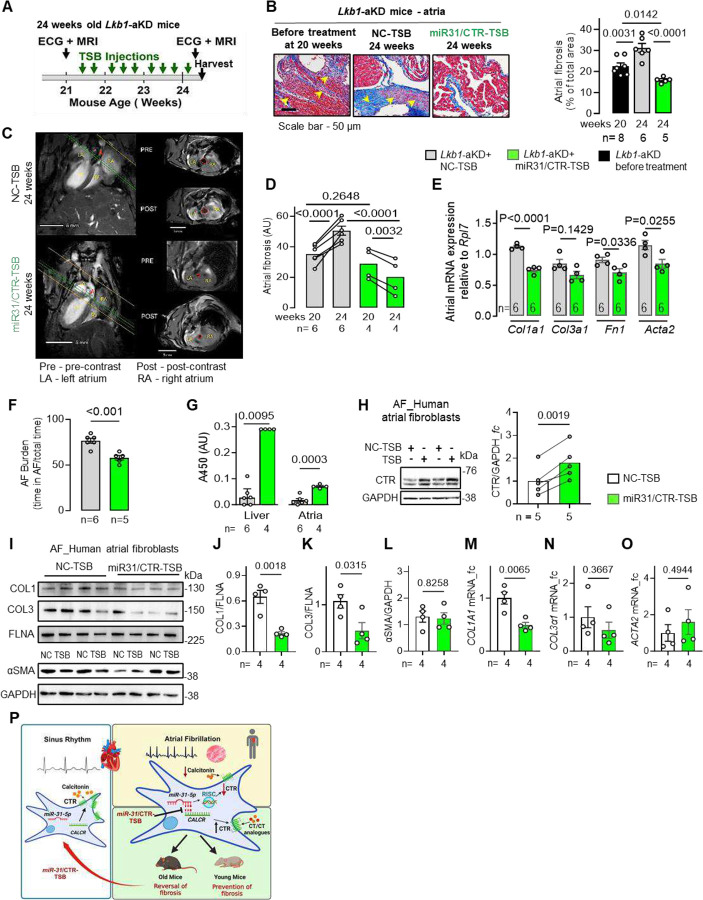
Selective blockade of the miR31/*Calcr* interaction with miR-31/CTR Target Site-Blocker (TSB) reduces established atrial fibrosis and arrhythmogenesis i*n vivo* in mice. **(A)** Experimental timeline in 24-week old *Lkb1*-aKD mice. **(B)** Atrial fibrosis (by Masson’s trichrome staining) in 24-week old *Lkb1*-aKD mice treated with *miR-31–5p*/CTR-TSB or NC-TSB control. **(C-D)** Effect of *miR-31*-5p/CTR-TSB or NC-TSB on atrial fibrosis (by MRI, assessed in the same animals at 20 and 24 weeks of age, pre- and post-TSB treatment respectively) in *Lkb1*-aKD mice. Representative MRI images from the 24-week post-TSB treatment; the orange and green lines indicate the area where the slices were acquired and the short-axis respectively; red circle outlines aorta. **(E)** Gene expression (by qPCR) of collagen 1 (*Col1a1),* collagen 3 *(Col3a1)*, fibronectin (*Fn1)* and *α*SMA (*Acta2*) in atrial tissue of mice post-*miR-31–5p*/TSB (or NC-TSB). **(F)** AF burden in *Lkb1*-aKD mice assessed between 20–24 weeks of age. **(G)** Hybridization assay for the post-TSB treatment levels of *miR-31–5p*/CTR-TSB (vs NC-TSB) in murine liver (positive control) and atrial tissue in 24 weeks old *Lkb1*-aKD mice. **(H)** Effect of *miR-31–5p*/CTR-TSB or NC-TSB on CTR protein in human ACFs. **(I-O)** Effect of *miR-31–5p*/CTR-TSB or NC-TSB on protein and mRNA levels of selected fibrotic markers, collagen-1 (COL1), collagen-3 (COL3) and alpha smooth muscle actin (αSMA) (normalised to filamin A, FLNA, or GAPDH as annotated) in ACFs from persAF patients. **(P)** Diagram depicting the main findings of the study. Data are expressed as mean ± SEM, except median and IQR (G - liver) P values are calculated by unpaired t test, except for one-way ANOVA (B), two-way RM ANOVA (D), on Mann-Whitney test (G - liver), paired t test (H); fc, fold change of control group; n, individual donors or mice.

While the prevention of atrial fibrosis was achievable in younger mice by therapy beginning before the development of fibrosis, we next questioned whether selective blockade of *miR-31*-5p/*CALCR* binding could exert a therapeutic effect on established atrial fibrosis (commonly observed in persAF patients) and arrhythmia. Thus, we subjected older *Lkb1*-aKD mice (20.5 weeks of age, with ~26% baseline atrial fibrosis, similar to the level observed in persAF patients) (29) to a 3.5-week treatment with *miR-31*/CTR-TSB, or NC-TSB (25 mg/kg, i.p., thrice per week) (fig. 5A). Atrial arrhythmias and fibrosis were monitored at baseline (20-weeks) and post-TSB treatment (24-weeks) by ECG and MRI (using a collagen-1 binding Gadolinium-diethylenetriamine pentaacetic acid dye, EP3533), respectively (29). Baseline atrial fibrosis did not differ significantly between groups; however, it decreased by ~30% (compared to the pre-treatment 20-week fibrosis levels) post-TSB treatment (confirmed by Masson’s trichrome staining - fig. 5B), while in the NC-TSB control group atrial fibrosis progressed by ~46% above the 20-week baseline fibrosis (P<0.0001) (Fig. 5C, D). The *miR-3*1/CTR-TSB-mediated reversal of atrial fibrosis was accompanied by a reduction in atrial mRNA expression of collagen-1 (by 33%, P=0.0001), fibronectin (by 22%, P=0.0336) and αSMA (by 26%, P=0.0255) with unchanged collagen-3 (fig. 5E) in TSB-treated (vs NC-TSB) mice. ECG revealed reduced AF burden (by ~16%; P= 0.033) in *miR-31*/CTR-TSB-treated mice (fig. 5F). A sandwich hybridization assay with a biotinylated LNA-capture probe showed efficient delivery of the *miR-31*/CTR-TSB to the atrial myocardial, with 4.2- and 8.1-fold increase (vs NC) in the atria and liver respectively (fig. 5G) (30). The *miR-31*/CTR-TSB treatment had no effect on blood pressure, heart rate, or cardiac contractility (**table S5**). In hACFs, the TSB-mediated selective inactivation of *miR-31*/CALCR binding approximately doubled CTR protein (P=0.0125) (fig. 5H) and suppressed mRNA and protein levels of collagen-1 (by ~68%; P=0.0018 and 51%; P=0.0065 respectively), reduced collagen-3 protein (but not mRNA) by 56% (P=0.0315) without changes in αSMA expression (fig. 5I–O). These observations confirm that the actions of *miR-31*/CTR-TSB in the *in vivo* mouse model are relevant to human atrium, and they demonstrate that selective deactivation of the miR-31/CTR signalling axis with miR-31/CTR-TSB reverses the established atrial fibrosis *in vivo*.

## DISCUSSION

Here, we identified *miR-31–5p* as a novel, upstream regulator of CTR signalling, whereby *miR-31–5p* binds the CTR-3’UTR to enhance its degradation/reduce translation and suppress CTR expression in human atrial fibroblasts, and promotes atrial fibrosis and arrhythmogenesis *in vivo*. Activation of this signalling cascade in the presence of heart disease, such as AF, or in a *miR-31-*Tg mouse model, induces fibrotic fibroblast activity leading to excessive atrial fibrogenesis and increased arrhythmia susceptibility. Patients with persAF, characterised by established atrial fibrosis and decreased CTR expression (13), have doubled *miR-31–5p* levels and increased *miR-31–5p*/*CALCR* interaction in ACFs. Animal studies showed that selective disruption of this signalling axis, with a *miR-31–5p*/CTR-TSB, was sufficient to prevent new (in young mice) and reverse advanced (in older mice) atrial fibrosis and arrhythmogenesis *in vivo* (fig. 5P).

A recently discovered endogenous, cardio-endocrine calcitonin signalling system in human atrial myocardium controls atrial fibrosis and arrhythmogenesis (13). Impaired CT-CTR signalling, as in patients with persAF, promotes atrial fibrogenesis and the formation of an arrhythmogenic substrate. While patients with persAF have suppressed CT production and CTR expression/internalization, they are unlikely to benefit from clinically approved CT-analogues unless CTR expression and function is restored. Furthermore, using CT-analogues is likely to further repress the already diminished cellular CTR surface expression as a result of ligand-induced and pre-existing (13) receptor internalisation and degradation, commonly observed with G protein-coupled receptors (31). Thus, in the light of the therapeutic potential of this signaling system for atrial fibrosis and arrhythmia, we searched for upstream mechanisms regulating CTR abundance in hACFs. We found that both right and left ACFs from patients with persAF have doubled levels of *miR-31–5p* (associated with ageing, and shown to play a role in ischemic and coronary heart diseases) (32, 33) that negatively correlated with CTR-mRNA (*CALCR*) expression, pointing towards a potential regulatory role for *miR-31–5p* on *CALCR* abundance. This was supported by the results of the reporter assay, which demonstrated changes in CTR gene/protein in hACFs after activation or suppression *miR-31–5p*, and by the confirmation of endogenous *miR-31–5p*/CTR-mRNA binding by RISC-pulldown in human AF-ACFs. Both *miR-31–5p* inhibition and selective disruption of *miR-31–5p*/CTR binding with TSB *in vitro* in human ACFs was sufficient, through restoring CTR expression, to repress some key fibrotic responses of ACFs, e.g., collagen production and proliferation.

Previous studies suggest transcriptional regulation of CTR expression (34). However, CTR mRNA expression is unchanged in AF patients (13), so transcriptional alterations are unlikely to play a major role in AF-hACFs in the intact human heart, despite *miR-31–5p*-mediated changes in CTR-mRNA expression in the *in vitro* studies using supra-physiological *miR-31–5p* levels. This discrepancy are likely due to a more complex endogenous environment and potential compensatory feedback in the endogenous myocardial environment of AF-patients.

While we found that *miR-31–5p* regulates *CALCR* abundance, it is common for a single gene to be regulated by multiple miRs (35); hence, CTR abundance in AF-ACFs might be controlled by other miRs. We tested the potential role of three other miRs previously documented to play a role in persAF that had a high predicted score for binding to CTR-3’UTR, but found no regulatory effects of those miRs on CTR. Notably, the logistic regression analysis showed that *miR-31–5p* was significantly associated with a higher risk of AF (Table S6, OD = 3.02, *P*-value = 0.025), suggesting that the upregulation of miR-31 in AF is not influenced by other confounding factors, highlighting its specific contribution to atrial fibrosis and AF pathogenesis.

*In vivo* studies confirmed the *miR-31–5p* mediated regulation of CTR in ffibroblast-targeted *miR-31*-overexpressing mice with a clinically relevant~2.5-fold (similar to that observed in patients with persAF) *miR-31–5p* upregulation. These mice had raised expression of both *miR-31–5p* and *−3p*, however, as latter has no binding site for CTR-3’UTR it was not studied further. Furthermore, given that a selective blockade of miR31–5p/CTR interaction with TSB successfully reverses atrial fibrosis and arrhythmogenesis indicates that this treatment is sufficient to preclude effects potentially mediated by actions via one of the other miR-31–3p targets. Notably, the changes observed in these mice were primarily driven by *miR-31–5p* produced by atrial (not ventricular) CFs, as ventricular-ACF fibrotic markers and LV-function did not differ between groups. Consistent with previous reports (16, 17), we observed greater levels of *miR-31–5p* in the atria, while it was barely detectable in ventricular myocardium (10-fold increase in atrial-CFs vs ventricular-CFs). Accordingly, we found that fibroblast-targeted *miR-31–5p* overexpression in mice caused an increase in atrial fibrosis associated with a significant prolongation of arrhythmic episodes and increased AF susceptibility. These changes seem to be independent of sex (we observed no differences between males and females), and developed in the absence of left atrial dilatation or changes in left ventricular systolic or diastolic function in *miR-31*/Cre mice. Therefore, a fibroblast-selective *miR-31* expression increase, comparable to that seen in patients with persAF, is alone sufficient to promote structural, fibrotic remodelling that translates into enhanced arrhythmia-maintaining substrate and vulnerability to AF. Notably, *miR-31–5p* has also been shown to regulate the electrical properties of atrial-CMs in persAF (16, 17); therefore, it is plausible that *miR-31–5p* (via distinct gene targets) represents a common master-driver of electrical and structural remodelling of the atrial myocardium in AF.

To test the relevance of the *miR-31–5p*/CTR-mRNA interaction to atrial remodelling, we utilized a previously characterised *Lkb1*-aKD mouse model of spontaneous AF (22). These mice (similar to AF patients) have 2-fold upregulated *miR-31–5p* and reduced CTR expression and, thus represent a clinically relevant (in terms of *miR-31*-5p/CTR signalling) model. Selective blockade of the *miR-31–5p*/*CALCR* interaction in mouse atria with miRCURY LNA (25) miRNA power *miR-31*/CTR-TSB, which was successfully delivered to the atrial myocardium, specifically (without altering the expression of other *miR-31* targets) increased atrial CTR expression, confirming an endogenous *miR-31–5p*/CTR interaction *in vivo*. Furthermore, 3-week TSB therapy in young (8-week-old) mice fully prevented the development of atrial fibrosis and significantly reduced both AF burden and duration. Although young mice displayed an atrial fibrotic burden (~6% of total area) comparable to that of non-transgenic age-matched mice, this does not reflect the typical clinical presentation of patients with established structural remodelling, like in persAF, who typically exhibit advanced (~22%) atrial fibrosis (28). Thus, we administered TSB (with higher frequency treatment regimen) in older (20-week-old) mice with established (~26%) atrial fibrosis. Pre- and post-treatment (at 20 and 24 weeks) MRI and histological assessment with Masson’s trichrome staining showed a reversal (by ~30% below the 20-week baseline) of atrial fibrosis in *Lkb1*-aKD mice *in vivo*. As in mice, *miR-31*/CTR-TSB treatment of human AF-ACFs suppressed production of some key fibrotic markers (e.g., collagen-1/−3). Hence, *miR-31*/CTR-TSB administration might offer a new approach to reverse advanced structural remodelling in the atrial myocardium, such as that encountered in patients with persAF. In a canine model of HF-induced atrial fibrosis and AF, complete reversal of HF does not lead to reversal of fibrosis and the AF substrate (36), so active therapy is likely needed to decrease already-established fibrosis. Given that *miR-31* upregulation in persAF is atrial specific (16) such therapy may help to circumvent off-target effects on ventricular myocardium, a common limitation of many current AF therapies (37). Furthermore, use of selective miR-mRNA-TSB agents may help to evade the concerns of off-target effects presented by global miR inhibition (37), e.g., changes in multiple (including physiologically important) *miR-31* targets identified by RNAseq. Encouragingly, *miR-31*/CTR-TSB administration had no effect on previously identified *miR-31* targets (e.g., *Nos1, Dmd, Stk40, Bap1*), confirming the high selectivity of the *miR-31*/CTR-TSB approach in hACFs and mouse atrial myocardium. Strikingly, 3-week TSB pulse-therapy was effective in not only preventing fibrosis from developing, but also reversed (by 30%) advanced atrial fibrosis *in vivo* in older *Lkb1*-aKD mice.

Given that ageing-/senescence-related processes (18–20), as well as ischemic (21) or coronary heart disease (24) can lead to *miR-31* upregulation, it is conceivable that *miR-31* upregulation in AF might be secondary to the underlying cardiac pathology, or ageing, rather than driven by AF itself. However, the latter cannot be excluded, as *miR-31* rises in *Lkb1*-aKD mice who display aging-related remodelling localised to atrial (but not ventricular) myocardium. Intriguingly, both upregulation of m*iR-31–5p* and impaired CT/CTR signaling are also linked to the age-associated processes in bones and joints, osteoporosis and osteoarthritis (19, 38–41). Thus, the role of *miR-31–5p*/CTR axis and the *miR-31–5p*/CTR blocking strategies may be of relevance and interest to the (patho)physiology of skeletal system.

We also noted that a prominent reduction in atrial fibrosis was associated with a relatively modest decrease in AF burden in older mice. This might be due to the relatively short (3.5 weeks) TSB treatment period or the insufficient CTR activation by its ligand CT, which is progressively suppressed in *Lkb1*-aKD mice. Thus, it is possible that a combined therapeutic approach of *miR-31*/CTR-TSB and hormonal CT replenishment (with clinically approved CT-analogues) might exert an even more potent anti-fibrotic and anti-arrhythmic effect. The results presented here, taken in the context of the existing evidence demonstrate the specificity of the miR31/CALCR-TSB approach and position CALCR as an important miR-31–5p downstream target in the context of fibrosis and arrhythmia. Together these data suggest enrichment of this miR in the atrial myocardium compared to LV. Furthermore, given that miR-31–5p is highly enriched in the atrial myocardium, strategies to manipulate miR-31–5p can offer primarily atrial-targeted therapeutic options, allowing to circumvent the non-specific effects on the ventricular myocardium – a serious limitation of the current antiarrhythmic drugs.

*In summary,* we have identified a novel *miR-31–5p*/CTR-mRNA regulatory mechanism upstream of the cardiac calcitonin system in human atrial myocardium. Furthermore, we found that advanced atrial fibrosis, commonly seen in persAF, is characterised by hACFs enriched for *miR-31–5p* which, via binding to the CTR-3’UTR, activates fibroblast proliferation and collagen production. Selective disruption of *miR-31–5p*/CTR-mRNA binding reversed atrial fibrosis and decreased AF burden *in vivo* in a clinically relevant model of progressive atrial fibrosis and arrhythmia. Thus, selective blockade of the *miR-31–5p*/CTR-mRNA interaction with *miR-31*/CTR-TSB treatment, potentially in combination with currently approved hormonal CT-therapy, may offer a safer, atrial-selective strategy to restore CT signalling and re-establish control over atrial fibrogenesis and prevent associated arrhythmia susceptibility in patients with a history of AF. The miR31/CTR-TSB offers a target-selective approach in miR therapeutics, which will have to be further validated in human AF in clinical trials.

## Supplementary Files

This is a list of supplementary files associated with this preprint. Click to download.

- EXCELFILEWB.xlsx
- SUPPLEMENTARYMATERIALS.docx

## CLINICAL PERSPECTIVE

### What is new?

- MicroRNA (miR)-31–5p is upregulated in atrial fibroblasts from AF patients and suppresses fibroblast calcitonin receptor (CTR) expression and signaling.
- Selective blockade of miR-31–5p/CTR-mRNA binding with a LNA-miR-Target-Site-Blocker increased mouse atrial CTR expression, reversed advanced atrial fibrosis and prevented atrial arrhythmogenesis in an in vivo model. These findings provide a proof of scientific principle for the importance of CTR downregulation by miR-31–5p, along with a potentially viable approach to reversing atrial fibrosis.
- This study represents the first example of an RNA-based therapeutic capable of reversing established atrial fibrosis that forms a substrate for AF’

### What are the clinical implications?

- The prevention of atrial fibrosis, a key component of the AF substrate in many patients, has been an elusive clinical target. Our findings provide evidence supporting the importance of the cardiomyocyte-calcitonin/fibroblast-CTR paracrine signaling axis in controlling atrial fibrogenesis and AF progression.
- Out findings reveal a novel miR-31 based control mechanism that is important in regulating atrial fibroblast function and AF pathophysiology.
- The effectiveness of selective blockade of miR-31–5p/CTR-mRNA binding in reversing atrial fibrosis and suppressing the AF substrate provides a viable target lead for the development of novel mechanism-based fibrosis-targeted AF therapies.
